# Draft genome of the scabies mite

**DOI:** 10.1186/s13071-015-1198-2

**Published:** 2015-11-10

**Authors:** S. Dean Rider, Marjorie S. Morgan, Larry G. Arlian

**Affiliations:** Department of Biological Sciences, Wright State University, 3640 Colonel Glenn Hwy, Dayton, OH 45435 USA

**Keywords:** *Sarcoptes scabiei*, Scabies mite, Genome sequence

## Abstract

**Background:**

The disease scabies, caused by the ectoparasitic mite, *Sarcoptes scabiei*, causes significant morbidity in humans and other mammals worldwide. However, there is limited data available regarding the molecular basis of host specificity and host-parasite interactions. Therefore, we sought to produce a draft genome for *S. scabiei* and use this to identify molecular markers that will be useful for phylogenetic population studies and to identify candidate protein-coding genes that are critical to the unique biology of the parasite.

**Methods:**

*S. scabiei* var. *canis* DNA was isolated from living mites and sequenced to ultra-deep coverage using paired-end technology. Sequence reads were assembled into gapped contigs using de Bruijn graph based algorithms. The assembled genome was examined for repetitive elements and gene annotation was performed using *ab initio*, and homology-based methods.

**Results:**

The draft genome assembly was about 56.2 Mb and included a mitochondrial genome contig. The predicted proteome contained 10,644 proteins, ~67 % of which appear to have clear orthologs in other species. The genome also contained more than 140,000 simple sequence repeat loci that may be useful for population-level studies. The mitochondrial genome contained 13 protein coding loci and 20 transfer RNAs. Hundreds of candidate salivary gland protein genes were identified by comparing the scabies mite predicted proteome with sialoproteins and transcripts identified in ticks and other hematophagous arthropods. These include serpins, ferritins, reprolysins, apyrases and new members of the macrophage migration inhibitory factor (MIF) gene family. Numerous other genes coding for salivary proteins, metabolic enzymes, structural proteins, proteins that are potentially immune modulating, and vaccine candidates were identified. The genes encoding cysteine and serine protease paralogs as well as mu-type glutathione S-transferases are represented by gene clusters. *S. scabiei* possessed homologs for most of the 33 dust mite allergens.

**Conclusion:**

The draft genome is useful for advancing our understanding of the host-parasite interaction, the biology of the mite and its phylogenetic relationship to other Acari. The identification of antigen-producing genes, candidate immune modulating proteins and pathways, and genes responsible for acaricide resistance offers opportunities for developing new methods for diagnosing, treating and preventing this disease.

**Electronic supplementary material:**

The online version of this article (doi:10.1186/s13071-015-1198-2) contains supplementary material, which is available to authorized users.

## Background

Scabies, a skin disease caused by the mite *Sarcoptes scabiei*, affects millions of humans worldwide and causes significant morbidity and discomfort. In chronic cases it can lead to hyperkeratosis (crusted or Norwegian scabies) coupled with secondary bacterial infections that may result in renal (glomerulonephritis) and heart (rheumatic fever) diseases [1–3]. In wild mammal populations scabies may cause isolated deaths or even significant mortality in a population. In domestic livestock such as cattle, goats, sheep, and pigs, scabies can result in reduced agricultural productivity (e.g. milk production, growth rates). Interestingly, these mites consume little oxygen (0.002 and 0.0008 μl O_2_/hr/female and male, respectively) so their energy demand on the infested host is small relative to the metabolic rate of the host [4]. This would suggest that the pathology in the host is the result of damage to the skin barrier, substances that the mite deposits in the host’s skin, the inflammatory and immune responses that the mite induces, and bacterial infections often associated with scabies infestations.

*S. scabiei* is an obligate parasite of humans and of more than 100 species in 27 families of domestic and wild mammals including rabbits, chamois, ibex, cheetahs, lions, wombats, gorillas, coyotes, foxes, wolves, dingoes and stray domestic dogs [5]. Scabies mites parasitizing the various host species are largely morphologically indistinguishable. Thus, it is unclear if the mites parasitizing different mammalian host species are different species or if they are strains or variants of the one species *S. scabiei*. It appears that some strains can permanently parasitize multiple host species (e.g., both dogs and rabbits) while some cannot, although cross-infestations among hosts has not been extensively studied [6–8]. Some temporary cross-infestations on different host species can last more than 10 weeks [8]. A recent molecular analysis based on the mitochondrial *cox*1 gene suggests that there are four distinct groups (species) of *S. scabiei* that parasitize humans [9]. Other studies suggest *Sarcoptes* mites from various hosts and different geographical locations consist of a single heterologous species [10–12]. Further molecular genetic studies based on a much larger set of gene sequences are needed to resolve the relationship between the strains/species of *S. scabiei* that parasitize the many mammalian species and if there are multiple genetically distinct species of these mites. Analysis of the scabies genome may help clarify the species question.

Currently, the preferred treatments for human scabies utilize the topical or systemic acaricides permethrin and ivermectin, respectively [13]. In addition to toxicity concerns, resistance to these acaricides is now documented in some populations, resulting in treatment failures [13]. Thus, new therapies for this disease are needed and detailed genetic information may elucidate the mechanisms of resistance and open avenues for the development of new treatments.

Little is known about the biology and host-parasite interactions of scabies mites. However, studies have shown that the mites produce substances that modulate some aspects of the host immune, inflammatory and complement reactions that allow the mites to at least initially survive and reproduce to become established in the host skin [14–27]. This includes influencing the secretion of cytokines and chemokines in epidermal keratinocytes and dermal fibroblasts [16, 17, 26], the expression of cell adhesion molecules from microvascular endothelial cells of blood vessels of the skin [25], inhibiting the activation of the complement pathways [14, 15, 28, 29], stimulating Interleukin-10 secretion from T-regulatory cells which down-regulates a T-helper cell-mediated immune response [21], skewing the balance between the Th1 and Th2 immune responses [19], and modulating the function of peripheral blood mononuclear cells [20]. Similarly, the phylogenetically-related, free-living house dust mites *Dermatophagoides farinae*, *D. pteronyssinus* and *Euroglyphus maynei* are the sources of molecules that modulate cytokine secretion and expression of adhesion molecules of dermal fibroblasts, epidermal keratinocytes, mast cells, basophils and bronchial epithelial cells of the airways and disrupts tight junctions [30–42]. The immune modulating molecules, genes controlling the synthesis of these molecules, and the mechanisms controlling expression of these genes in these related mites have not been elucidated.

Scabies and house dust mites are the sources of many antigenic molecules that induce humoral immune responses in humans. Some of the antigens from these species are cross-reacting and serum antibodies built to scabies mites recognize antigens from house dust mites and vice-versa [43–46] and vaccination with a house dust mite whole body extract induced some protection from scabies infestation [46]. The proteins and antigenic determinants responsible for this cross-reactivity are largely unknown. However, several cloned *S. scabiei* peptides have high homology with antigens from the dust mites [47, 48]. Both scabies and house dust mites are placed in the Hyporder Astigmata [49]. Genomic data may provide the tools to further understand the cross-reactivity among these related mites and facilitate the development of a diagnostic test and vaccine for scabies that have long been confounded by these cross-reactivity issues.

Studies show that scabies mites are attracted to body odor and body warmth and that the mites prefer specific skin locations [50–52]. The sensory physiology and mechanisms associated with responding to carbon dioxide, host odor, host body temperature, and skin lipids have not been elucidated. Likewise, the properties of the host that influence the selection of one host species over another and that subsequently govern the selection of preferred burrowing sites in specific locations have not been illuminated. How factors such as cytokines, chemokines, skin lipid composition, serum and blood components (e.g. phagostimulants such as ATP) influence feeding and reproduction also remain to be determined. Genomic sequence data and molecular studies may provide a basis for answering these questions.

Here we report the completion of a draft of the *S. scabiei* genome using a strain of scabies mites that was originally obtained from infested dogs. The origin of the infestation on the source dogs was unknown, however, this strain permanently infests New Zealand white rabbits and scabies in wild rabbits is common [5, 10, 53–55]. The sequence of the scabies genome may provide some of the tools needed to investigate many of the unknowns discussed above that relate to the scabies mite survival, reproduction, host-parasite interactions and may facilitate studies in these areas, the development of a diagnostic test for scabies, new treatments, and a vaccine for protecting against this disease.

## Methods

The strain of *S. scabiei* that was chosen for genome sequencing (var. *canis)* is an inbred strain that has been maintained as an isolated laboratory culture for over thirty years (>700 generations; [7]) under Animal Use Protocol (AUP) #981, approved by the Wright State University Laboratory Animal Care and Use Committee. *S. scabiei* var. *canis* mites were surface sterilized as previously described [16]. Genomic DNA was isolated from living mites of all active stages using the Wizard SV genomic DNA purification system (Promega, Madison, WI) and the manufacturer’s animal tissue protocol. To enable proper digestion of mite tissues using this kit, mites were ground in digestion buffer using a Dounce homogenizer. All steps leading up to the overnight proteinase K digestion were performed on ice to mitigate endogenous DNase activity. TruSeq Library construction and paired end sequencing were done by Beckman Coulter Genomics (Danvers, MA) to generate ~114 million paired reads (2x150 bp; ~57 million fragments; 350 bp insert size).

**Fig. 1:**
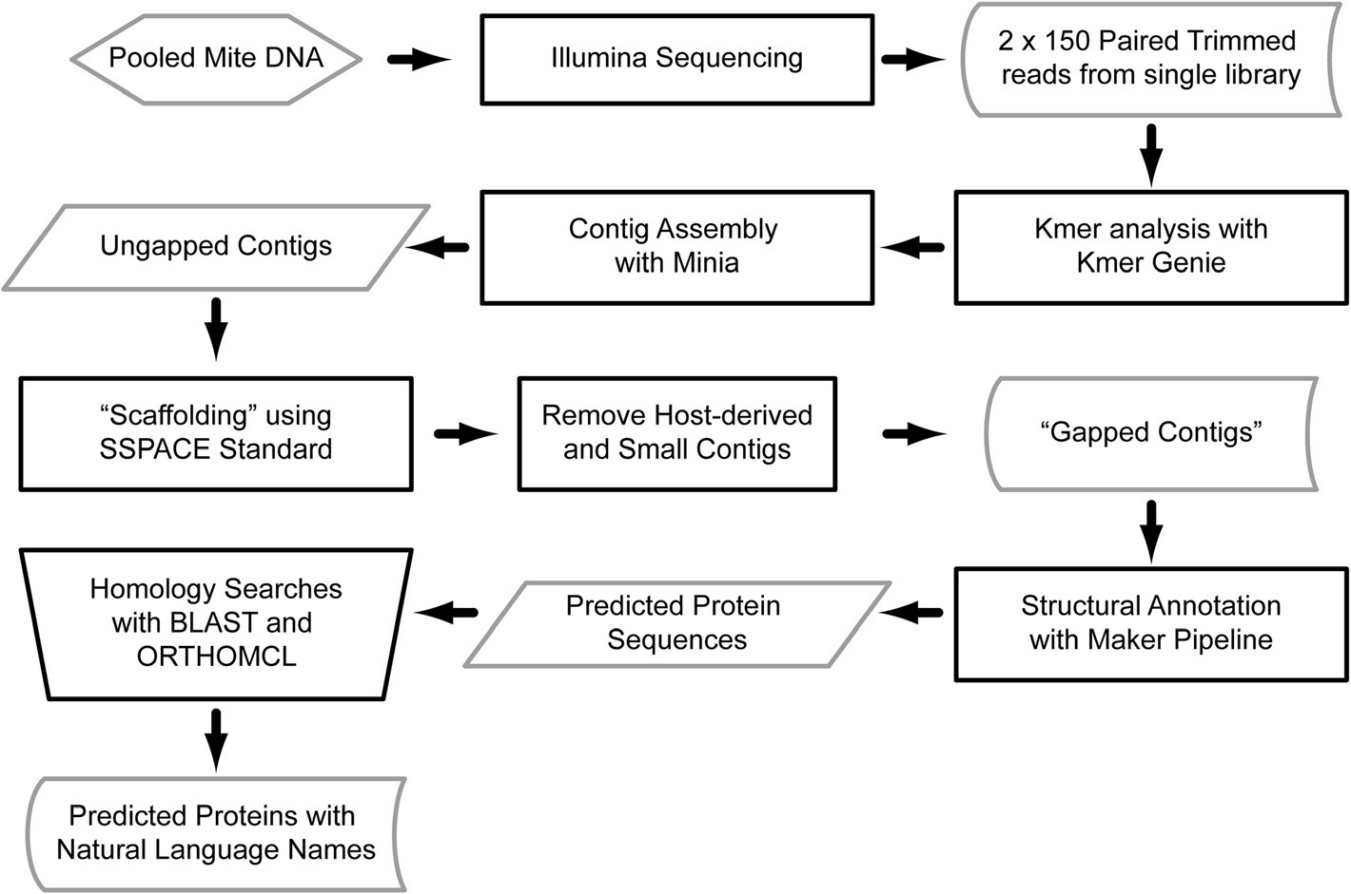
Genome assembly and annotation processes and outputs are indicated in the flow chart. Sequence Reads, Gapped Contigs, and Predicted Proteins are all stored in public databases under BioProject PRJNA268368

The genome assembly and annotation pipeline are indicated in Fig. 1. Unless specifically indicated, the software programs were used with the recommended or default settings. To estimate the best value of k and the minimum abundance for a given Kmer when using De Bruijn graph assembly algorithms, we used KmerGenie (v1.6741; [56]). The best estimate provided was k = 63. However, this value represented a local maximum, followed by one additional peak at k = 71, and a shoulder that dropped substantially after k = 101. Thus, different values of k were used to generate multiple assemblies. Minia (v1.6088; [57]) was used with SSPACE-STANDARD (v3.0; [58]) to assemble trimmed reads into gapped contigs. Within SSPACE, Bowtie (v0.12.5; [59]) was used to map reads during “scaffolding” and without implementing contig extension. After correcting for adapter trimming, the paired end library size provided to SSPACE was 372 +/- 334.8 bp. The best assembly (largest N50 and maximum contig size) was generated using k = 71, a minimum Kmer abundance of 16, and an initial genome size estimate of 98 megabases. The gapped contigs were filtered to remove mammalian sequences, and sequences less than 200 nt. The remaining contigs were examined by BLAST [60] for the presence of extranuclear genomes (mitochondria, symbionts) using the following as queries: mitochondrial DNA (NC_012218), Wolbachia (AE017196), Enterobacter (CP001918). The contigs were also examined by BLAST using the Representative_genomes databases (05/28/2014 update available at ftp.ncbi.nlm.nih.gov/blast/db/). Contigs that matched with 75 % or greater query coverage and an e value of 1 x 10^−4^ or less were considered suspect.

The core eukaryotic genes mapping approach (CEGMA) was used to test genome completeness with 248 highly conserved eukaryotic loci [61]. BLAST was also used with an extended CEGMA set of 458 proteins (http://korflab.ucdavis.edu/datasets/cegma/core/core.fa). Gapped contigs were also examined by BLAST for expressed sequence tags and other *S. scabiei* nucleotide sequences that were present in public databases. The Benchmarking Universal Single-Copy Orthologs (BUSCO) strategy was also used to test the completeness of our assembly and gene set using the arthropod, metazoan, and eukaryote profiles [62].

The MISA perl script [63] was used to scan the genome of *S. scabiei* and other assembled Acari genomes for simple sequence repeats of (1 nt with >10 copies, 2 nt with >6 copies, 3 nt with >5 copies, 4 nt with >5 copies, 5 nt with >5 copies, 6 nt with >5 copies, 7 nt with >5 copies, and 8 nt with >5 copies). The other genomes examined were: *Achipteria coleoptrata* GCA_000988765.1, *Dermatophagoides farinae* GCA_000767015.1, *Hypochthonius rufulus* GCA_000988845.1, *Ixodes scapularis* GCA_000208615.1, *Metaseiulus occidentalis* GCA_000255335.1, *Platynothrus peltifer* GCA_000988905.1, *Steganacarus magnus* GCA_000988885.1, *Tetranychus urticae* GCA_000239435.1, and *Varroa destructor* GCA_000181155.1.

Maker software was implemented with the accessory software programs RepeatMasker [64], SNAP [65], and Augustus [66]. During software training, SNAP utilized the ixodesB hidden Markov model (HMM) and Augustus used the Nasonia HMM. Scabies expressed sequence tags (ESTs), and all proteins in the protein database (downloaded from NCBI in September 2014) for the spider mite *Tetranychus utricae*, the tick *Ixodes scapularis*, the western predatory mite *Metaseiulus occidentalis*, and the social spider *Stegodyphus mimosarum* were used in training. A hidden Markov model (HMM) for scabies was generated for SNAP, and was used in the final round of annotations. Hints for these gene predictions came from 1,041 EST sequences for *S. scabiei,* 17,091 EST sequences from other Sarcoptiform mites (NCBI EST database), and the reference proteomes of *Dermatophagoides farinae* (provided by Stephen Kwok-Wing Tsui, The Chinese University of Hong Kong), *Metaseiulus occidentalis* (NCBI Refseq), *Tetranychus urticae* (Ensemble Metazoa Release 23), *Drosophila melanogaster* (Uniprot), *Pediculus humanus corporus* (Uniprot), *Tribolium castaneum* (Uniprot), and the CEGMA core 458 proteins indicated above. Over three hundred of the Maker predictions possessed introns less than 10 bp. These protein predictions were passed through the GeneWise-2.4.1–14 [67] to help identify alternative intron boundaries which were adopted in the final annotation. Annotations of the mitochondrial genome were aided by the MITOS web server (http://mitos.bioinf.uni-leipzig.de/index.py) and RNA Weasel (http://megasun.bch.umontreal.ca/RNAweasel/).

Reciprocal best blast hits were identified using Legacy BLAST and the perl script orthoparahomlist.pl [68] and output was used to estimate the number of proteins that are shared between *S. scabiei* and other Acari with annotated genomes (*Ixodes scapularis*, *Tetranychus utricae, Dermatophagoides farinae,* and *Metaseilus occidentalis).*

Predicted protein identities were generated using a combination of methods to identify orthologs or homologs in other organisms, including best reciprocal blast hits against the Refseq proteins from *Metaseiulus occidentalis* and *Ixodes scapularis*, submission of predictions to OrthoMCL [69] for clustering, and name suggestions from best BLAST hits using the PLAN database [70] using default settings. Preliminary identities were compared against the results of BLAST against the Refseq proteomes of *Homo sapiens*, *Drosophila melanogaster*, *Pediculus humanus*, and *Tribolium castaneum*, as well as local RPS blast searches of the proteins for conserved domains from the Pfam, NCBI-curated domains, and Smart data sets. Construction of RPS profile databases used the suggested threshold of 9.82 and scale of 100.0 while BLAST searches utilized default settings. An e-value cutoff of 1 x 10^−4^ was used to eliminate poorly matching candidates from BLAST results. BLAST results were then examined for consistency (best hits with similar names from two or more proteomes and the presence of similar conserved domains) and manually edited. With few exceptions, proteins that did not have a high scoring match to a conserved domain or proteins from other organism were labeled as ‘hypothetical protein’. Proteins with matches that were discordant were also labeled as hypothetical proteins. Proteins with partial or complete matches to conserved domains, but for which inconsistent results were obtained from the Refseq proteome BLASTs were provided names based on the domains they contained (e.g., ‘ankyrin repeat containing protein’). Proteins that showed consistent results from Refseq BLASTs and RPS BLASTs inherited names based on their similarity to putative homologs (e.g., ‘tubulin gamma-1-like’).

To identify candidate immunomodulatory proteins, proteins from scabies mites and other organisms (mites, ticks, mosquitoes, lice, etc.) that have known or suspected immunomodulatory properties were used as seeds in BLAST searches against the genome and predicted proteome of *S. scabiei*. Phylogenetic comparisons among proteins relied on ClustalW alignments [71] and either Neighbor joining trees [72] or bootstrapped maximum likelihood trees [73] that used the Jones Taylor Thornton evolution model [74] with 5 gamma substitution rates. Phylogenetic reconstructions were implemented with Mega6 software [75].

## Results

### Genome assembly metrics and estimates of genome completeness

Contigs assembled by Minia had an average depth of coverage of 174x and totaled 60.2 megabases (Mb). After implementing SSPACE to build scaffolds (aka “gapped contigs”) based on paired end data, filtering out potential contaminants (human and rabbit), and filtering out contigs smaller than 200 bp, the draft genome assembly (with gaps) was 56.2 Mb. While this value is much lower than the anticipated 98 Mb previously reported for this species, the existing analyses of genome size utilized PCR-based methods and displayed considerable variability among replicate samples [76]. However, the assembly appears to be reasonably complete (see below). The contig N50 for the filtered assembly was 11.6 kb, but contigs found to possess predicted coding regions were generally larger than non-coding contigs. This may reflect a reduced tolerance within coding regions for repetitive sequences that fragment the genome assembly. This assembly included a mitochondrial genome contig. Statistics for the assembly at different steps in the process are given in Table 1. The raw data, gapped contigs, and annotations were deposited into public databases under BioProject PRJNA268368. Excluding 5′ and 3′ untranslated regions, roughly 27.7 % of the genome was covered by protein coding genes, with 21.8 % representing coding sequence. A typical gene in *S. scabiei* had an average length of 1461 bp, with three exons and two introns. Exons averaged 370 bp, while introns averaged 149 bp.

**Table 1:** Statistics for the *S. scabiei* genome at various stages of the assembly

	Minia	SSPACE	Filtered	Coding
Assembly size (Mb)	60.2	59.2	56.2	38.5
Number of contigs	62,434	36,298	18,600	3,569
N50 (kb)	2.9	10.5	11.6	22.2
Largest contig	100.5	358.8	358.8	358.8

To estimate genome completeness, we examined the assembly for highly conserved protein coding genes that are found in nearly all eukaryotes using the CEGMA analysis software [61]. CEGMA indicated 93.55 % genome completeness based on the 248 loci included in the test. We expanded on this using BLAST to examine an updated set of 458 CEGMA loci. All queries had matches (at least in part) in the genome assembly. With this expanded gene set, ~91 % of loci were considered to be complete (at least 70 % query coverage). BUSCO, a similar strategy for the quantitative assessment of genome completeness, was also used [62]. This approach likewise makes use of genes that are expected to be single copy and query sets have been developed for representative taxonomic groups: eukaryotes, metazoans, and arthropods. Of the genes anticipated to be present as single copies in an eukaryote, the benchmarking strategy indicated that the *S. scabiei* predicted gene set contained roughly 92 % of examined loci (83 % complete genes and 8.8 % fragmented genes) out of the 429 queried. Benchmarking performed poorly when the arthropod set was used: 66 % present of 2675 queried (55 % of which were complete). The arthropod set is largely based on insects and similarly low scores were obtained for other annotated Acari genomes (reported in supplementary data from [62] and Additional file 1). This reflects a need for a benchmarking query set tailored to the Acari. We queried the assembly for all scabies nucleotide sequences that were present in public databases. The assembly had matches to over 96 % of the available scabies ESTs, and 98 % of other scabies nucleotide sequences (the ESTs that did not match were low complexity sequences). This suggested that our assembly represented over 95 % of the *S. scabiei* genome.

### Extranuclear genomes

The scabies mitochondrial genome was identified as a single 13.6 kb contig in the assembly. The mitochondrial genome contained 13 protein coding loci and 20 transfer RNAs (tRNAs). The mitochondrial protein coding genes as well as most of the tRNA genes were in the same order and with the same orientations that are present in the mitochondrial genome from the pyroglyphid house dust mite, *Dermatophagoides pteronyssinus*. The *D. pteronyssinus* mitochondrial genome has an unusual gene arrangement compared to most arthropods [77]. Thus, the colinearity between the *S. scabiei* mitochondrial DNA and that of *D. pteronyssinus* is consistent with previous analyses indicating that house dust mites likely evolved from a lineage of obligate animal parasitic mites [78, 79]. Transfer RNAs for tyrosine and alanine were not identified in the *S. scabiei* mitogenome. Instead, the cysteine tRNA in *S. scabiei* occupied the space within a cluster of tRNAs where the alanine tRNA is located in *D. pteronyssinus*. Additionally, the position of the *S. scabiei* valine tRNA between the two mitochondrial rRNA genes was more similar to the deduced ancestral arthropod mitochondrion [80].

Commensal gut microbes and endosymbionts are common among mites (see [81] for a recent review). We used pooled, whole live mite bodies for nucleic acid isolations, which provided an opportunity for us to identify any symbiont genomes represented in our data. We examined the assembled contigs for the presence of *Wolbachia, Enterobacter* and other microbes from the non-redundant representative genomes database. While we did not find evidence for the presence of endosymbionts, three contigs contained sequences similar to, but not identical to the 23 s rRNA genes from *Corynebacteria* species. The three contigs were small and totaled 3.1 kb. The largest contig (2.5 kb) also had portions which did not match *Corynebacteria.* Thus, horizontal gene transfer cannot be ruled out. However, *Corynebacteria* are prevalent in the environment, on the skin of humans and laboratory animals, and are used for industrial-scale production of nucleotides [82–84]. The absence of additional corynebacterial sequences suggested the 23 s sequences could be contaminants, rather than originating from the mite or a commensal microbe. The lack of symbiont genomes in our data set was consistent with previous analyses indicating that *Wolbachia* is absent from *S. scabiei* [85], and that bacterial-derived endotoxin (lipopolysaccharide) levels are low in whole body *S. scabiei* extracts (unpublished data).

### Repetitive elements

**Fig. 2:**
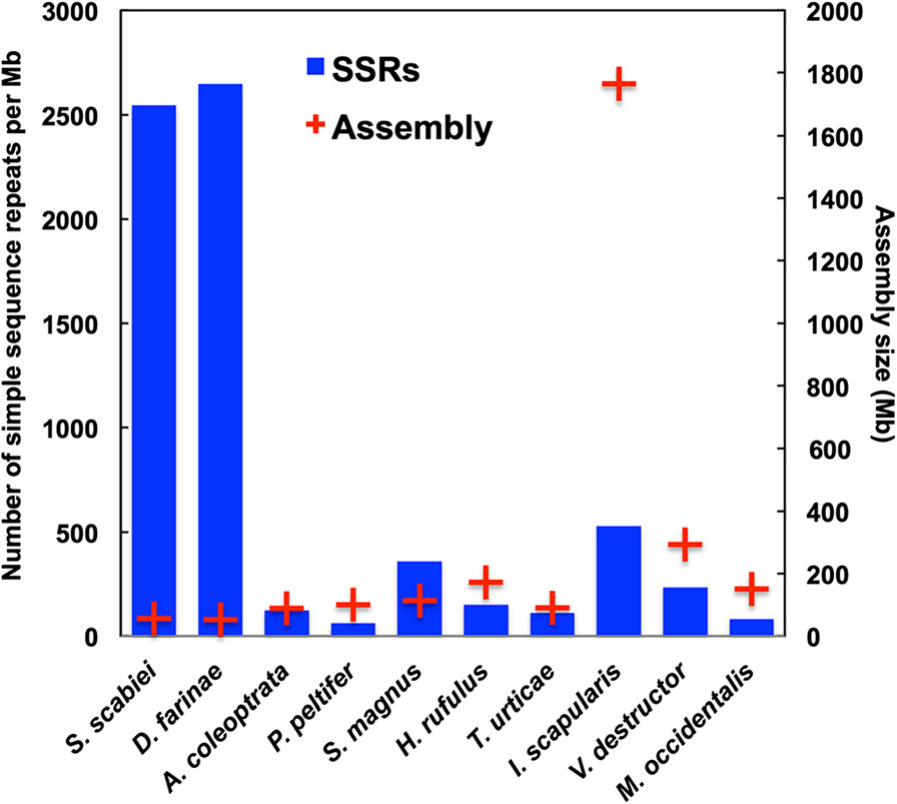
Number of simple sequence repeats (SSRs) per Mb for various Acari genomes. Assembly sizes are shown for comparative purposes

**Fig. 3:**
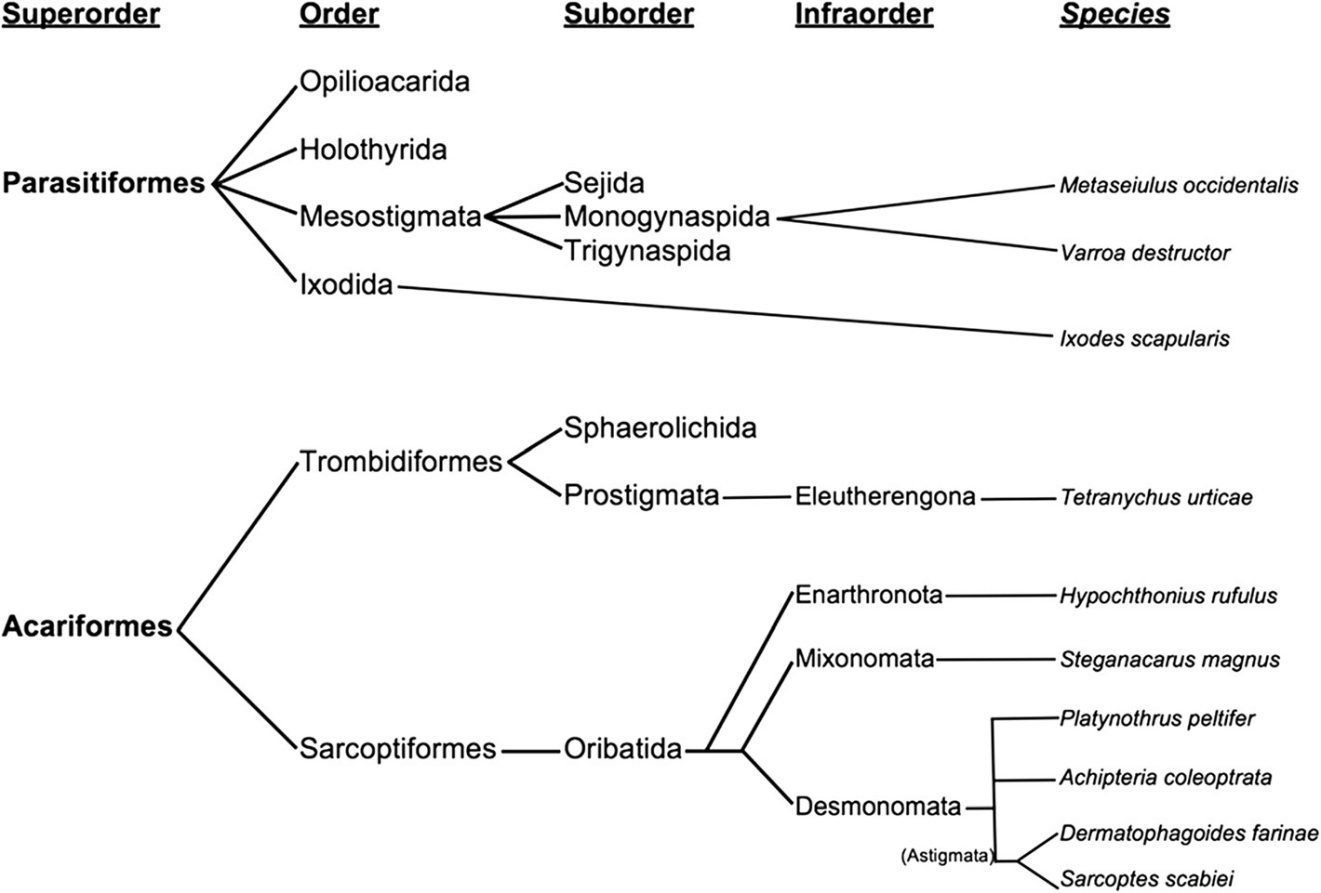
Phylogenetic relationship of the ten Acarine species whose genomes have been assembled. Constructed based on classifications from [49]

Simple sequence repeats (SSRs or microsatellites) have been used for genetic studies on *S. scabiei* to determine host-parasite associations, identify the sources of infestations, and to understand population structures within and between hosts [86–94]. We identified 142,638 microsatellite loci that possessed perfect repeats and these repeats accounted for ~3 % of the genome. In comparison with other Acari, the number of SSR loci was very high (Fig. 2). While the number of Acari genomes is limited to less than a dozen available assemblies, we found that the two mites in the Astigmata hyporder shared similarly small assembled genome sizes and large numbers of SSR loci. Despite having larger genomes, the number of SSR loci present in other Acari was low (Fig. 2). Within the Parasitiform mites (Fig. 3), the abundance of SSR loci showed a strong positive correlation with genome size (r squared 0.999) but among Acariform mite genomes, this correlation did not exist (negative correlation, r squared 0.393). The vast majority of SSR loci were present in intergenic regions or introns of *S. scabiei*, and only 1495 SSR were identified in the predicted coding regions. Approximately 90 % of the SSR sequences in the assembly were represented by (A/T)n, (AG/CT)n, and (ATC/GAT)n repeats. Within predicted coding regions, triplet repeats were the most abundant type of SSR, but (ATC/GAT)n, (A/T)n, (AAC/GTT)n were the most common individual repeat types identified. While it is not presently clear how these repeats evolved in Astigmatid mites, some may be the result of replication slippage, aberrant recombination and repair events, or transposable element activity at select locations in the genome [95]. All three major classes of transposase proteins were identified in the genome assembly by TEseeker [96] and RepeatMasker [64]. The most common types of repetitive elements other than SSR and low complexity regions included hundreds of fragments related to mobile DNAs (e.g. EnSpm, Maverick), LTR (eg Gypsy and Copia), LINE, RC, and SINE elements (Additional file 2 contains a complete count of element types identified by RepeatMasker). We also interrogated the assembled contigs for TTAGG telomeric repeats using BLAST and found at least sixteen contigs that possessed imperfect repeats of this sequence (these were not identified in the scan for SSR). While not definitive, this suggested that *S. scabiei* may possess and utilize archetypal telomeres.

### Predicted proteome

**Fig. 4:**
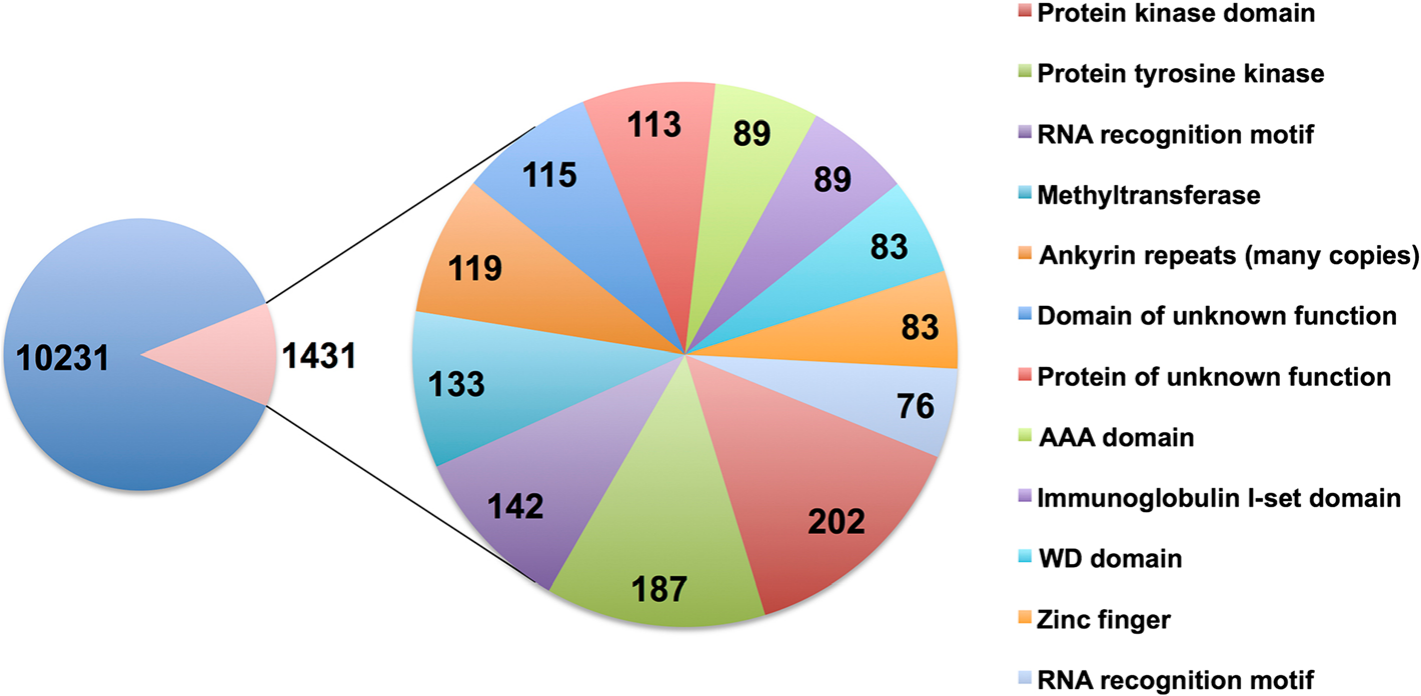
The most abundant Pfam domain descriptions in the predicted proteome of *S. scabiei.* The total number of Pfam domains exceeds the number of predicted proteins since some proteins contain multiple domains

**Fig. 5:**
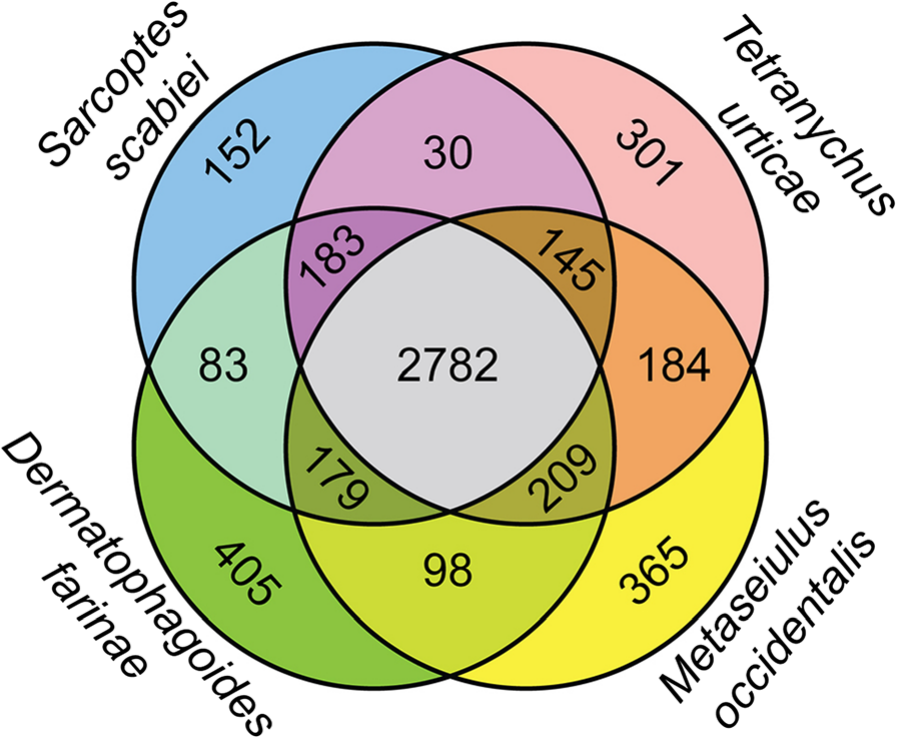
Venn diagram showing the relationships among Pfam domain types identified in the predicted proteomes of four mite species. This diagram is based on the presence or absence of a given domain without regard for its relative abundance within the proteome

The predicted proteome of *S. scabiei* was relatively small compared to the other available annotated Acari genomes, and contained 10,644 proteins (Table 2). The largest predicted protein-coding gene was 34 kb in length and encoded the titin/twitchin protein homolog (9021 aa, 1013.6 kDa). Analyses of the predicted proteome for conserved domains indicated that protein kinases and RNA binding proteins were abundant (Fig. 4). An examination of Pfam domains present in the predicted proteins from *S. scabiei* and three other mites showed substantial overlap in the types of protein families that are present across all four species (Fig. 5). The Pfam domain types that are shared across all four species displayed little variation in abundance based on Tukeys IRQ test for outliers. However, among the few domain types that displayed dramatic differences, the number of lipocalins (pfam00061) was about ten-fold higher in *Tetranychus urticae* than in the other mite species (Additional files 3 and 4). It remains unclear if these potential allergens correlate with the unique allergic responses to *T. urticae* that are experienced by farm and greenhouse workers [97, 98].

**Table 2:** Comparison of genome size, assembly size and number of predicted proteins for all Acari species for which data are available [76, 118–121]

	Genome size (Mb)	Assembly size (Mb)	No. of protein coding genes
*Sarcoptes scabiei*	96	56	10,644
*Dermatophagoides farinae*	Unreported	53	16,376
*Tetranychus urticae*	75	90	18,423
*Ixodes scapularis*	2,300	1,765	20,474
*Metaseiulus occidentalis*	89	151	11,444

**Fig. 6:**
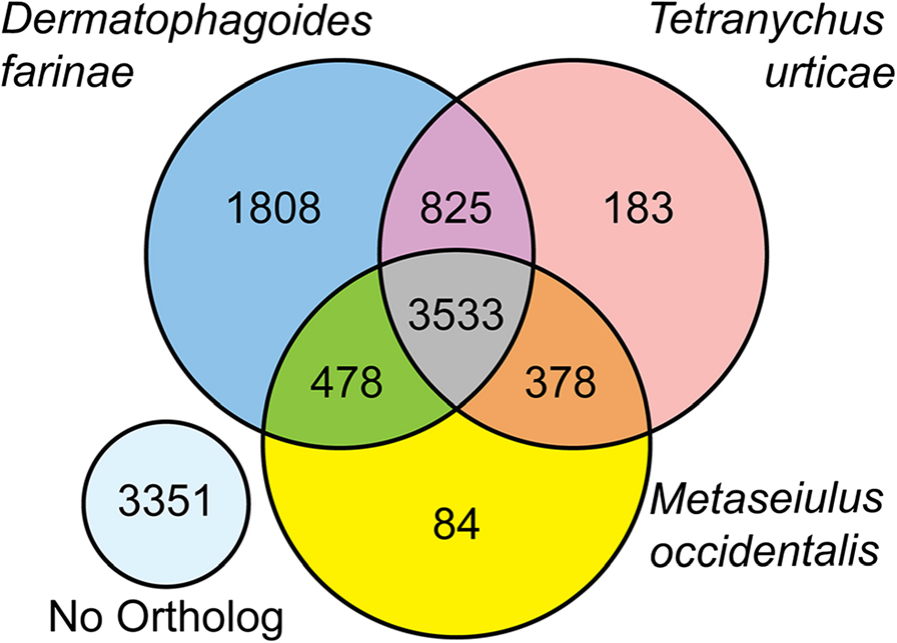
Venn diagram depicting numbers of predicted *S. scabiei* proteins with or without orthologs in three other mite species. Proteins with ortholog groups from other mite species also displayed substantial overlap with one another

Orthology relationships between *S. scabiei* proteins and those of other Acari indicated that ~67 % of the *S. scabiei* proteins possessed an ortholog in one or more of the annotated Acari genomes used for comparison. The number of orthologs shared between the two Acariform mites (*S. scabiei* and *D. farinae*) was the greatest (Table 3), with the members of the Parasitiformes showing similar numbers of potential orthologs that were shared with *S. scabiei*. In general, the number of shared orthologs among the Acari is consistent with their phylogenetic relationships (Fig. 3). Over 3,500 proteins were shared across the four mite species (Fig. 6). One of the largest protein families identified in *S. scabiei* posseses many members that do not have clear orthologs in other mites. However, all members of this protein family share a novel conserved region of unknown function that is also present in proteins from other mites, including the dust mite protein DFP-2. Outside of the conserved region, the proteins are highly divergent, which limited the identification of clear orthologs. The genes for these proteins are scattered throughout the *S. scabiei* genome and many of the family members encode proteins with secretion signals. The function of these proteins is unknown.

**Table 3:** Number of orthologs shared between annotated Acari genomes based on reciprocal best BLAST of predicted proteins

	*S. scabiei*	*D. farinae*	*T. urticae*	*I. scapularis*	*M. occidentalis*
*Sarcoptes scabiei*	10,644				
*Dermatophagoides farinae*	6,644	16,376			
*Tetranychus urticae*	4,919	4,937	18,423		
*Ixodes scapularis*	4,650	4,754	5,086	20,474	
*Metaseiulus occidentalis*	4,473	4,497	4,890	5,688	11,444

### Allergen homologs

At a molecular level, allergens are represented by a relatively small number of protein families [99, 100] and we examined the predicted *S. scabiei* proteome for allergen candidates. *S. scabiei* possessed homologs for most of the characterized group 1 through group 33 dust mite allergens (Table 4). Notable exceptions included the group 4 amylase, an antimicrobial peptide and the two related small structural proteins (group 5 and group 21). However, amylase protein coding genes that were not allergen homologs were present in the genome. Two tropomyosin candidates were identified (group 10). One of the tropomyosin genes, as well as an arginine kinase gene, was split between two contigs, and it is likely that other loci may be similarly fragmented. Among the three chitinase-like mite proteins that act as allergens, a clear homolog of the group 12 chitinase was not identified. However, two candidates for the group 15 and group 18 chitinases were identified. While one of the *S. scabiei* chitinase candidates displayed homology specifically to the group 15 allergen, the other chitinase allergen candidate showed homology to both the group 15 and group 18 allergens. This candidate had slightly better percent coverage for the group 18 allergens. While no molecular data appear to be available concerning the group 17 calcium-binding EF hand proteins, at least five EF hand domain containing proteins were identified by RPS blast against the Pfam domain database. Homologs for groups 1, 3, 6, 8, and 9 were present, and most resided in gene clusters (see below). Candidates other than those related to mite allergens were also present, including a vespid venom v5 homolog, two cupin domain-containing proteins, and four additional lipocalin-like proteins.

**Table 4:** Number of predicted house dust mite allergen homologs identified in the *Sarcoptes scabiei* genome

Allergen group	Biochemical function	No. of homologs
1	Cysteine protease	>10
2	Lipid binding protein	1
3	Serine protease	>18
4	α-Amylase	0
5	Structural protein	0
6 & 9	Serine protease	1
7	Lipopeptide binding protein	2
8	Glutathione-S-transferase	5
10	Tropomyosin	2
11	Paramyosin	1
12	Possible chitinase	0
13	Fatty acid binding protein	1
14	Apolipophorin	1
15	Chitinase	1
16	Gelsolin	1
17	Calcium-binding EF-hand protein	5
18	Chitinase	1
19	Antimicrobial peptide	0
20	Arginine kinase	2
21	Structural protein	0
22	Lipid binding protein	1
23	Chitin binding protein	1
24	Ubiquinol-cytochrome c reductase binding protein	1
25	Triose phosphate isomerase	1
26	Myosin, light chain	11
27	Serpin	10
28	Heat shock protein	8
29	Cyclophilin	12
30	Ferritin	3
31	Cofilin	1
32	Inorganic pyrophosphatase	1
33	α-Tubulin	2

Allergen group and biochemical function information from [98]

### Allergen gene clusters

Expressed sequence tags from *S. scabiei* [47, 48, 101–103] have revealed that at least three allergen groups are represented by multi-gene families, including the cysteine proteases (group 1), serine proteases (groups 3, 6 and 9) and the glutathione S transferases (group 8). Many of the cysteine and serine protease gene family members identified in *S. scabiei* var. *hominis* appear to encode inactivated (mutated) versions of the enzymes which interfere with the host complement system [15, 102, 103].

Eight of the estimated twenty cysteine protease homologs identified in the *S. scabiei* var. *canis* genome were present in a gene cluster at the end of a 76 kb contig (JXLN01010058.1). These gene family members were in a head-to-tail orientation within a 16 kb region next to a casein kinase I gamma-like gene that is oriented in the opposite direction. The presence of closely spaced, but highly similar gene paralogs like the proteases proved to be problematic for the gene prediction algorithms, and resulted in a number of gene fusions which required manual re-annotation. Two more cysteine protease gene homologs were identified in another contig of 7.9 kb. Each of the cysteine protease homologs is highly divergent, and not all are clear orthologs of those previously reported in *S. scabiei* var. *hominis*. The organization of the genes within the cluster suggests that mutation of the active site cysteine to serine (present in a subset of the homologs), as well as other potential inactivating mutations (premature stops, for example), has occurred independently of the gene duplication events.

At least 50 serine proteases appeared to be encoded by the *S. scabiei* genome, and a subset of those serine protease-like proteins were related to the group 3, 6, and 9 dust mite allergens. Phylogenetic comparisons of the predicted proteins indicated that the majority of the allergen-like serine proteases were more closely related to the group 3 allergens, than to the group 6 or group 9 allergens. The group 6 and group 9 allergens from other mites formed a clade within the serine proteases, and only one *S. scabiei* protein was present at the base of that group. The remainder of the serine protease allergen candidates displayed affinity to the group 3 allergens. Among the group 3 allergen homologs, genes for fifteen serine protease homologs were arranged in a head-to-tail orientation within a 24 kb region next to a putative transcription factor on a 30 kb contig (JXLN01017869.1). The contig terminated in the middle of one paralog, suggesting that, although the adjacent contig was not readily identified, the gene cluster may be even larger.

Similarly, four of the five mu-type glutathione-S-transferase genes (group 8 allergen candidates) were located in a cluster on a 14.9 kb contig (JXLN01017505.1). These genes did not contain introns, and the nearest gene (a potassium channel gene) was ~7 kb away.

A substantial fraction of known allergen homologs with host immune modulating functions appear to be the result of local gene duplications in *S. scabiei.* Additional mutations have apparently also resulted in significant differences in the sequences and numbers of cysteine and serine proteases present in the two lineages that lead to *S. scabiei* var. *canis* and *S. scabiei* var. *hominis*.

### Other immunomodulatory molecules

Most ecto—and endo-parasites have evolved multiple mechanisms to evade or manipulate their host’s inflammatory, complement, innate or adaptive immune systems. Often, the molecules involved are derived from salivary gland or gut secretions, and a wealth of information is available on salivary and gut proteins from ticks, mosquitoes and other hematophagous arthropods [104–107]. A small number of these proteins have also been used as vaccine candidates in attempts to prevent infestations and block disease transmission (particularly for ticks). Despite having divergent lifestyles and being taxonomically distant, the data from other organisms provides an opportunity to seek out candidate immunomodulatory pathways encoded in the *S. scabiei* genome.

We used publicly available compilations of tick sialoprotein [107] and mosquito sialotranscriptome [108] data sets to identify candidate salivary gland protein homologs. However, several thousand candidates were identified. We then chose to focus on candidates based on those that are reported to have antigenic properties [109], are vaccine candidates [110–112] or represent known immunomodulatory pathways [113–115]. Over 300 candidates, including a few select vaccine candidates were identified with this approach (Additional files 5 and 6). Candidates included new members of the *S. scabiei* macrophage migration inhibitory factor gene family, which have been demonstrated to be involved in diverse host-parasite interactions [27, 116–118]; multiple tetraspanins that are involved in cell adhesion, migration and proliferation; [119], an Angiotensin-converting enzyme; and leukotriene A-4 hydrolase. The enzymatic pathways for producing both microsomal and cytosolic prostaglandin E2 also appeared to be present. Thus, as in ticks, salivary gland exocytosis may be regulated by prostaglandins in *S. scabiei*. Salivary-derived prostaglandins and leukotrienes may also serve to modulate the host immune response and lower the host’s threshold for histamine-induced pruritus.

## Discussion

There is a dearth of genomic sequence data for the Acari. Assembled genomic data for the Acari are limited to ten species (Fig. 3). The genomes for *Achipteria coleoptrata*, *Hypochthonius rufulus*, *Platynothrus peltifer, Steganacarus magnus*, and *Verroa destructor* have been assembled but have not been annotated. Annotated genomes exist for only five species, *Ixodes scapularis* [120], *Metaseiulus occidentalis* [121], *Tetranychus urticae* [122], *Dermatophagoides farinae* [123] and now *S. scabiei*.

Phylogenetically, the Acari with annotated genomes are very distantly related (Fig. 3). The tick, *I. scapularis* and the mite *M. occidentalis* are placed in the Superorder Parasitiformes. The other three genera *Tetranychus*, *Sarcoptes*, and *Dermatophagoides* are placed in the Superorder Acariformes, however, *Tetranychus* is in the order Trombidiformes and suborder Prostigmata while *Sarcoptes* and *Dermatophaoides* are in the order Sarcoptiformes and suborder Oribatida and are closely related Astigmatid mites. Therefore, the parasitic ticks belong to the Superorder Parasitiformes while the *S. scabiei* parasite is in the Superorder Acariformes, a very distant relationship.

These species of Acari represent diverse life styles. The *S. scabiei* annotated genome provides data for a non-blood feeding permanent obligate parasite of the epidermis of the skin of mammals. It has a very different biology and host-parasite interaction compared to ticks that are obligate but temporary blood-feeding parasites and from the other species of mites that are not parasites of mammals. The *S. scabiei* genome allows for comparison of genomes from two obligate blood/plasma feeding parasitic Acari that can modulate aspects of their hosts’ innate and adaptive immune systems (*I. scapularis* and *S. scabiei*), as well as comparison of the *S. scabiei* genome to that from a plant-feeding parasitic mite that sucks fluids from the leaves of host plants (*T. urticae*), an ectoparasitic mite that sucks hemolymph from the host honey bee (*V. destructor*), a predaceous mite that feeds on other mites (*M. occidentalis*), a free-living mite that feeds on stratum corneum from the skin epidermis after it is shed (*D. farinae*), and several free-living soil mites. Comparison of genes of these mites that have different life styles, biology and classification that places them in distant or similar taxa, may identify interesting gene profiles or sets of genes that are unique as well as common to the mites in these various taxa.

Genomic data provide the tools to facilitate study of many aspects of the scabies mite biology, evolution, and host-parasite interactions. These may include:

1. Clarifying the phylogenetic relationships and evolution of scabies species or strains that infest different host species.
2. Clarifying the phylogenetic relationships of scabies mites within the Acari and particularly among the Astigmata.
3. Determining the molecular basis and mechanisms for host preference.
4. Predicting protein production and function including predicting proteins responsible for immune/inflammation modulation and cross-reactivity between scabies and house dust mites. Some of these proteins may be candidates for vaccine or diagnostic test development.
5. Identifying genes that are predicted to code for antigenic salivary, molting and digestive enzymes. This could lead to the cloning of these genes for screening as candidates for vaccines or diagnostic tests.
6. Identifying genes that are responsible for resistance to the current acaricides of choice for treatment of scabies (permethrin and ivermectin) and the mechanisms responsible for this resistance and screening genes for potential new targets for novel acaricides (e.g., growth/development inhibitors, molting inhibitors, ovicides).
7. Identifying genes that control the production of proteins (e.g., inhibitors of the inflammation in the skin) that may be candidates for novel biopharmaceuticals that can be used to treat other skin diseases such as psoriasis, eczema and atopic dermatitis.

## Conclusion

These scabies genomic data provide essential tools for researchers seeking to develop methods to effectively prevent, treat and control this disease. In addition, these data provide tools to facilitate study of the phylogeny, evolution, and host-parasite interactions, including modulation of the host’s innate and adaptive immune systems.

## Additional files

Additional file 1:
**BSUCO analysis metrics for Scabies and other acari genomes and gene sets.** (XLSX 6 kb) Additional file 2:
**Abundance of repetitive element types identified by RepeatMasker.** (XLSX 115 kb) Additional file 3:
**All Pfam hits in the scabies proteome with 1 x 10** ^**−4**^ **cutoff.** This is a redundant list (since some proteins contain multiple domains) that can be used to count the number of specific Pfam domains in the proteome. (XLSX 358 kb) Additional file 4:
**Normalized abundance of shared Pfam domains in four mite proteomes.** Values represent the number of domains that would be present in a proteome bearing 10,000 Pfam domains. Blue represents minor outliers that are higher than expected and Red represents minor outliers that are lower than expected. There were no major outliers detected. (XLSX 115 kb) Additional file 5:
**Locations and putative identities of scabies homologs for genes which are immunogenic and present in the salivary glands of ticks and are listed in** **[** 107 **].** (XLSX 17 kb) Additional file 6:
**Locations and putative identities of scabies homologs for vaccine candidates listed in** **[** 110 **].** (XLSX 5 kb)
